# Intrinsically disordered domains deviate significantly from random sequences in mammalian proteins

**DOI:** 10.1186/1471-2105-11-S7-S7

**Published:** 2010-10-15

**Authors:** Shunsuke Teraguchi, Ashwini Patil, Daron M Standley

**Affiliations:** 1 Laboratory of Host Defense, WPI Immunology Frontier Research Center (IFReC), Osaka University, 3-1 Yamadaoka, Suita, Osaka 565-0871, Japan; 2 Human Genome Center, Institute of Medical Science, The University of Tokyo, 4-6-1 Shirokane-dai, Minato-ku, Tokyo 108-8639, Japan; 3 Laboratory of Systems Immunology, WPI Immunology Frontier Research Center (IFReC), Osaka University, 3-1 Yamadaoka, Suita, Osaka 565-0871, Japan

## Abstract

**Background:**

In order to characterize mammalian intrinsically disordered domains (IDDs) we examined the patterns in their amino acid abundance as well as overrepresented local sequence motifs. We considered IDDs from mouse proteins associated with innate immune responses as well as a set of generic human genes. These sets were compared with artificially generated random sequences with the same overall amino acid abundance and length distributions. IDDs were then clustered by amino acid abundance, and further analyzed in terms of co-occurrence of clusters with functionally characterized Pfam domains.

**Results:**

Overall, IDDs were very different from randomly generated sequences. The deviation from random distributions was at least as great as that for ordered domains, for which the deviation can be rationalized in terms of strong evolutionary pressure for structure and function. The co-occurrence of certain Pfam domains with specific IDD clusters was found to be significant (p-value < 0.01). Local sequence motifs that were over-represented in the innate immune set consisted mostly of low complexity fragments, primarily characterized by amino acid repeats, and could not be assigned an obvious functional role.

**Conclusions:**

Our results suggest that IDDs are constrained within a narrow subset of possible sequences. This is most likely a result of biophysical restraints that have yet to be elucidated. More detailed examination of the functional relationship between the IDDs and associated Pfam domains is one possible avenue of investigation.

## Background

Intrinsically disordered domains (IDDs) are abundant in eukaryotic proteomes [1], especially in cell signaling proteins [2]. It has been shown that many IDDs become ordered upon binding other macromolecules [3], and that their binding modes can be diverse [4] with the length of the IDD modulating the binding affinity [5]. This allows them to function as hubs in protein-protein interaction networks [6-8].

IDDs can be recognized by their amino acid composition, which is biased toward hydrophilic residues [9]. There is also a bias toward low-complexity regions, characterized by the abundance of one or a few amino acids, in IDDs [10]. IDDs have been shown to evolve more rapidly than ordered domains [11,12], while maintaining their length and location in the protein [8]. Despite their functional importance, IDDs are generally filtered out when performing structure-based protein function prediction in order to focus attention on better-characterized ordered domains.

In this study, we determined the levels of similarity between IDDs in a set of mammalian immune and non-immune proteins and compared them to random and ordered sequences. IDDs were then clustered into similar sequence groups for proteins in the immune and non-immune sets and their associated ordered domains were studied. We further extracted common sequence motifs in the IDDs in an attempt to identify common sequence patterns.

## Results

### Overview

We examined two sets of eukaryotic IDDs to study their sequence similarity and their association with specific functional domains. The first was taken from a set of 1580 mouse genes relevant to macrophage response to microbial stimulation (Innate Immune Set). The second is from a set of 1663 human proteins selected at random, excluding those in the Innate Immune Set. The use of two sets afforded the opportunity to examine whether, after filtering obvious homologs, there was greater similarity within a set of functionally related proteins than within a set of proteins picked at random. For the purposes of our analysis we here define IDDs as any predicted disordered segment of 30 residues or more, using the program Disopred2 [1] at a false positive threshold of 5%.

Since sequence repetitions are abundant in IDDs, similarity measures that do not require pair-wise alignment are convenient. To this end, frequency distributions were computed for single and multiple amino acid occurrences within an IDD, as described in Methods. Since dissimilarity measures, such as Kullback-Leibler divergence or the Student's t-test are sensitive to the sample size, we chose instead to directly measure the similarity between two frequency distributions using two methods. The first is a variation of a Gaussian-based score that has proven useful in structure comparison (eqn. 2 in Methods). The second is by explicit enumeration of all possible amino acid sequence motifs of length 2-5. Using the latter method, we examined the ratio of observed to expected frequencies for each possible motif and discuss those that deviate significantly from their expected values.

### Disordered sequences are not random

**Figure 1:**
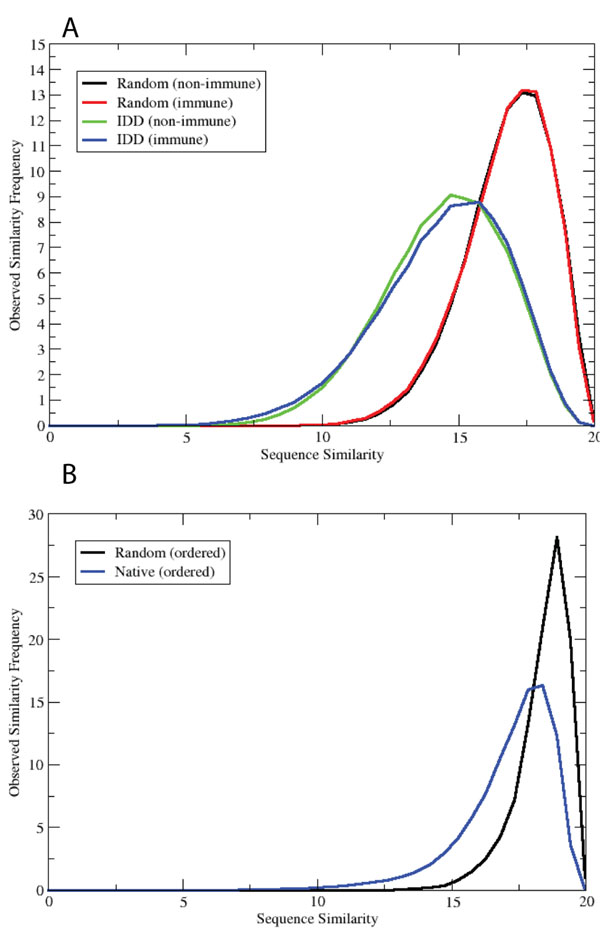
**Observed frequency of amino acid histogram similarity scores**. The similarity score is scaled from 0 to 20 for convenience (i.e., 100% identical histograms would have a score of 20). *Native *refers to actual protein sequences and *random *to artificially generated sequences with the same overall amino acid composition and length distribution as native sequences. A) Data are shown for non-immune random, immune random, non-immune IDD, and immune IDD sets. B) Data are shown for random ordered and native ordered sequences.

Using the Gaussian-based similarity score we carried out all-against-all comparison of IDDs in the immune and non-immune sets. For each of the IDD sets, we also constructed a randomized sequence set with the identical overall amino acid composition, sequence number and domain length frequency by shuffling the residues in the original native sequence set, as described in Methods. We then constructed a histogram of the similarities within each of the resulting 4 sets by binning the calculated similarity scores into 50 equal-sized windows. As figure 1A illustrates, the random distributions are skewed toward the high end of the similarity spectrum, while native IDDs are much more diverse. Thus, the similarity between either of the IDD sets is much lower than the similarity between random-immune and random-non-immune sets. This shows clearly that IDDs are not constructed randomly from a pool of disorder promoting amino acids. As a comparison, we performed the same calculations on a set of ordered protein sequences extracted from a representative set of structured domains. As figure 1B illustrates, the ordered domains are also much more different from each other than random sequences are, even when the length distribution and overall composition are held constant. However, the overall similarity (as indicated by the peak in the distribution) is much lower in the disordered set than in the ordered set.

**Figure 2:**
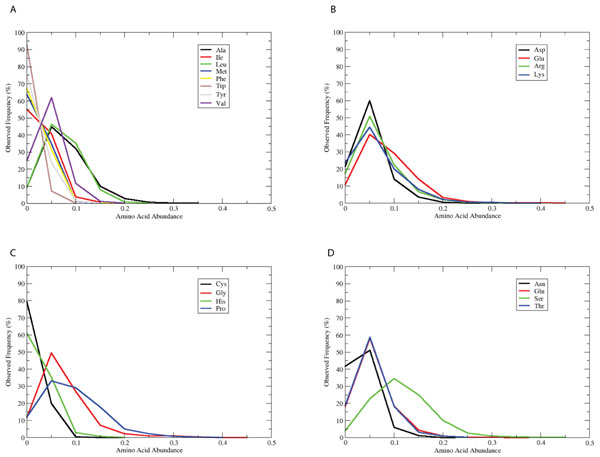
**Observed frequency of amino acid abundances in generic IDDs**. The abundance of each of the 20 amino acids was computed from the generic IDD set, and then displayed as a normalized (percentage) histogram. The plots are grouped together as A) Hydrophobic; B) Charged; C) Other; D) Polar.

As figure 2 illustrates, the frequency distributions of individual amino acids within IDDs are in general non-symmetric. That is, there are long tails to the right indicating that some sequences are rich in a particular amino acid type. As has been well documented by others, these particular amino acids include Glu, Gly, Pro, and Ser [9]. When examined closely, we observe broad peaks for Glu, Gly, and Pro at large abundances (25-30% of the IDD length). This non-uniform distribution of amino acids in IDDs is consistent with the non-random similarity distributions described above.

### Randomizing IDDs predicted to reduce IDD content

Disorder prediction was performed on the randomized IDD sequences from the non-immune set. The percentage of disordered residues was predicted to decrease by 45% overall (data not shown) upon randomization. This result also suggests that IDDs are not just randomly aligned regions but have some specific tendency to be biologically constrained. However, we must make this conclusion with caution, since it may be due to an artifact of the prediction algorithm: Since the Disopred2 program was trained on native sequences, it is not clear whether it is justified to interpret the results when applied to randomized sequences. However, we can at least say that the Disopred2 program correctly identifies the difference between the random sequences and the real IDDs. Based on this result, we speculate that true IDDs have requirements beyond mere disorder; namely, the ability to fold upon encountering a target protein or the need to contain specific local sequence patterns necessary for biochemical function.

### Sequence motifs in IDDs

In order to identify motifs in IDDs we enumerated all possible sequence fragments from 2-5 amino acids in length and examined the frequency distributions in the IDD and random sets. We computed the ratio of observed frequencies to their expectation values by calculating the frequencies of each single amino acid in the dataset.

**Figure 3:**
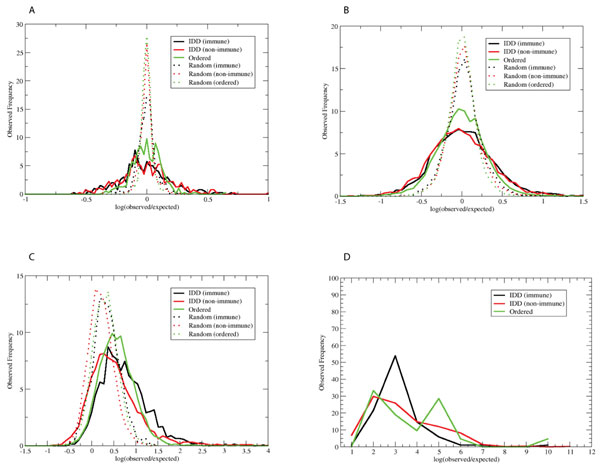
**Sequence motif analysis**. All possible fragments of length 2-5 were enumerated and their observed and expected frequencies were computed. The x-axis represents the natural log of the ratio of the observed to the expected frequency. The y-axis is the histogram of these values in 6 different sets: Immune IDDs, non-immune IDDs, ordered domains, random immune IDDs, random non-immune IDDs, and random ordered domains. Panels A-D illustrate motifs of length 2-5, respectively. Random sequences produced zero counts for the motifs of length 5.

Since fragments with very rare occurrence could not be interpreted statistically, we discarded any motifs with less than 10 counts. Figure 3 displays the histogram of the distribution of the natural log of the ratios. It shows that the deviations of observed frequencies for IDDs from the expected values are larger than that for randomly generated sets, indicating that IDDs tend to have some particular motifs. In principle, the center of the distribution is 0, where the observed frequency equals the expectation value. This is actually the case for doublet and triplet fragments where almost every sequence motif is observed a number of times. However, for the quartet and quintet, the center is shifted to the right, most likely due to the fact that motifs with less than 10 counts were discarded. For all the random sets, there was no Quintet with observed frequency greater than 10. (Note that this simple model does not distinguish between multiple motifs found in the same protein sequence and those found in different sequences.)

We next examined the specific motifs that were over-represented in the innate immune set. From table 1, we can see that the over-represented motifs consist almost entirely of low complexity fragments that contain at least 3 occurrences of the same amino acid. We could not easily assign a functional role to such motifs. For example, there are no obvious SH3, SH2, or kinase binding sites on this list. However, a large number of amino acid repeats were found in these motifs.

**Table 1:** Local motifs over represented in the immune set.

Motif	Ratio	Immune/Non	Motif	Ratio	Immune/Non	Motif	Ratio	Immune/Non
GSPGP	1.58	9.5/6.0	PGPGL	--	15.0/0.0	APAAA	--	20.3/0.0
EDEEE	1.4	42.3/30.2	DEEEE	1.8	59.3/33.0	RGRGR	2.78	109.8/39.4
AAEAP	--	17.7/0.0	PGQPG	--	19.0/0.0	EEAEE	--	17.8/0.0
PPLPP	1.3	16.0/12.3	PGGPG	--	17.6/0.0	PPPLP	1.29	15.2/11.8
AAAEA	--	22.8/0.0	EDEED	1.7	56.4/33.2	PGPGG	1.65	14.6/8.9
PPPPL	1.35	15.2/11.2	EEEGE	1.7	18.5/10.8	SSASS	2.16	7.1/3.3
GSGSG	2.16	17.5/8.1	PAAPP	1.88	12.3/6.5	KKKKK	1.68	184.1/109.7
QPPPP	1.26	19.2/15.2	EEDEE	2.33	48.0/20.6	EEEDD	1.56	51.7/33.2
EEGEE	--	18.5/0.0	PAPAP	1.47	14.3/9.8	TTTTT	--	176.8/0.0
EAPAA	--	19.3/0.0	PGLPG	1.55	19.5/12.5	PPVPP	1.58	18.4/11.7
APAPA	1.6	13.2/8.2	SGSGS	--	11.3/0.0	QQQQP	1.29	71.7/55.4
GPGGP	--	19.0/0.0	EPEPE	--	15.8/0.0	PPPPQ	1.32	18.2/13.8
PPPPA	2.05	22.3/10.9	QQQQQ	1.34	1239.8/926.7	PAPQV	--	31.5/0.0
LPPPP	1.4	20.2/14.4	SSTSS	1.37	8.2/6.0	PQPPP	1.47	13.9/9.4
EEEEE	1.28	211.9/165.8	PLPPP	1.39	12.6/9.1	PPAPP	1.26	11.1/8.8
SSCSS	--	31.3/0.0	AEAPA	--	25.7/0.0	EEEED	1.91	73.4/38.5
PEPEP	--	14.8/0.0	EEEEV	1.53	34.9/22.9	DDEEE	--	56.4/0.0
QQPPP	1.65	29.5/17.9	GRGRG	2.65	84.4/31.9	QQQPP	1.31	38.3/29.2
EEEDE	1.27	36.7/28.9	KGEKG	--	38.1/0.0	GEEEE	1.68	25.9/15.3
GPPPP	1.45	12.4/8.5	PAAAE	--	19.3/0.0			

The criterion for including motifs in the table was that the ratio of observed over expected was higher in the immune set than in the non-immune set by a factor of at least 1.25.

### Clustering IDDs by amino acid composition

In order to characterize IDDs with similar amino acid compositions, we carried out hierarchical clustering by average linkage with respect to a Gaussian-based similarity score described in Methods (eqn. 2). The number of clusters was monitored as a function of the similarity cutoff threshold (data not shown) and all 4 sets showed a similar sensitivity to the cutoff value. A clustering cutoff value of 0.15 was chosen as few new clusters were formed for moderately larger values. Note that the clustering cutoff value represents the average value of eqn. 3 over the entire cluster. These clusters are used in the following comparison between innate immune and non-immune sets.

### Innate immune and non-immune sets contain similar IDD clusters

**Figure 4:**
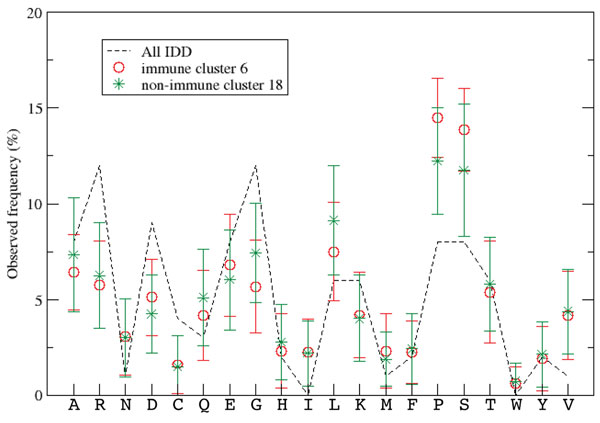
**Histogram clusters**. An example of similar histogram clusters in the Immune (6) and non-immune (18) sets, corresponding to rows 8-11 in Additional File 1. Error bars correspond to standard deviations from the mean; the background distribution (all sequences) is indicated by the black dotted line.

In order to identify IDD clusters that appeared in both the immune and non-immune sets the Gaussian-based similarity score was generalized to compare IDD clusters, rather than pairs of IDD sequences as performed in the previous section. We defined the similarity between two amino acid frequencies in two clusters as the similarity between their average values and we set the square of the width of the Gaussian *w^2 ^*to the sum of the square of the standard deviations (eqn. 4). With this formulation of similarity, clusters from the immune set were paired with clusters from the non-immune set by maximizing the Gaussian similarity score. An example of two similar clusters in comparison with the background distribution of amino acid frequencies is shown in figure 4. The amino acid frequencies for the IDD clusters identified are listed in Table 2 showing differences in the abundance of amino acids across clusters.

**Table 2:** Amino acid frequency distributions of similar IDD clusters

Cluster	A	R	N	D	C	Q	E	G	H	I	L	K	M	F	P	S	T	W	Y	V
Immune (0)	11	6	2	5	1	5	14	4	2	2	12	7	2	1	5	10	4	0	1	4
Non-Immune (99)	7	5	4	5	1	8	14	3	2	3	13	10	2	1	2	7	5	0	2	5

Immune (1)	8	5	2	5	1	5	7	11	2	2	7	5	2	2	12	11	6	1	2	4
Non-Immune (18)	7	6	3	4	1	5	6	7	3	2	9	4	2	2	12	12	6	1	2	4

Immune (3)	5	6	4	5	1	5	6	7	3	3	8	6	2	2	7	15	5	1	2	5
Non-Immune (41)	6	6	4	6	2	6	8	5	3	3	6	4	2	3	8	17	5	1	2	5

Immune (6)	6	6	3	5	2	4	7	6	2	2	8	4	2	2	15	14	5	1	2	4
Non-Immune (18)	7	6	3	4	1	5	6	7	3	2	9	4	2	2	12	12	6	1	2	4

Immune (9)	6	5	3	5	2	5	6	7	3	2	7	4	2	2	9	19	7	1	2	4
Non-Immune (23)	5	6	4	6	1	4	6	6	2	3	10	5	2	3	5	19	6	0	2	4

Immune (12)	7	5	3	5	1	6	13	6	3	2	9	4	2	1	15	7	4	0	2	6
Non-Immune (2)	8	6	2	5	1	4	9	7	2	2	8	6	2	2	15	10	5	1	2	4

Immune (15)	18	5	3	4	1	8	7	7	1	3	10	5	2	1	5	8	5	0	1	7
Non-Immune (126)	17	6	3	3	1	4	8	6	2	3	8	5	2	2	11	7	5	1	1	6

Immune (29)	7	6	4	6	1	5	11	6	2	3	8	8	3	2	6	9	5	1	2	5
Non-Immune (26)	5	6	4	6	1	5	9	5	2	4	9	8	2	3	7	10	6	1	2	4

Immune (36)	6	7	4	4	1	12	11	4	2	3	12	9	2	2	4	8	4	0	1	3
Non-Immune (30)	4	5	4	4	2	13	10	6	2	2	11	11	2	2	5	5	4	0	3	6

Immune (38)	7	4	3	4	2	4	6	7	2	1	9	4	2	3	20	10	4	1	2	5
Non-Immune (31)	6	4	3	6	1	5	4	6	3	2	9	3	2	2	20	12	6	1	1	4

Immune (41)	5	4	4	10	1	5	16	6	2	4	7	7	2	1	6	11	4	0	2	4
Non-Immune (38)	6	5	4	6	2	5	14	5	2	4	7	6	2	2	6	12	6	1	2	5

Immune (53)	5	6	4	4	2	5	10	7	2	3	5	4	2	2	8	11	7	1	2	8
Non-Immune (26)	5	6	4	6	1	5	9	5	2	4	9	8	2	3	7	10	6	1	2	4

Immune (56)	7	9	3	3	2	5	4	4	2	3	12	5	2	2	13	11	6	0	1	5
Non-Immune (18)	7	6	3	4	1	5	6	7	3	2	9	4	2	2	12	12	6	1	2	4

Immune (72)	8	9	4	4	1	5	7	5	3	3	6	4	2	2	7	12	10	1	1	4
Non-Immune (55)	8	11	3	4	1	4	9	6	3	2	8	5	2	2	7	13	5	1	2	4

Immune (79)	7	5	4	9	1	4	8	5	3	3	9	4	4	4	8	10	5	1	2	5
Non-Immune (26)	5	6	4	6	1	5	9	5	2	4	9	8	2	3	7	10	6	1	2	4

Immune (80)	11	6	1	4	0	9	10	5	1	3	4	13	2	2	15	7	5	1	1	3
Non-Immune (2)	8	6	2	5	1	4	9	7	2	2	8	6	2	2	15	10	5	1	2	4

Immune (94)	6	4	3	5	2	6	8	5	2	3	6	17	3	2	7	9	5	1	2	5
Non-Immune (42)	6	5	4	5	1	4	9	7	2	4	8	15	2	2	5	6	6	1	2	5

Immune (100)	4	6	3	7	2	13	14	6	2	3	6	5	2	1	9	7	6	0	1	5
Non-Immune (38)	6	5	4	6	2	5	14	5	2	4	7	6	2	2	6	12	6	1	2	5

Immune (107)	4	4	3	6	1	5	16	7	2	2	9	2	2	2	9	13	6	0	2	6
Non-Immune (38)	6	5	4	6	2	5	14	5	2	4	7	6	2	2	6	12	6	1	2	5

Immune (115)	6	4	3	5	1	4	9	6	2	4	6	11	2	2	11	10	7	1	1	4
Non-Immune (26)	5	6	4	6	1	5	9	5	2	4	9	8	2	3	7	10	6	1	2	4

Immune (175)	9	3	2	4	1	6	7	8	3	2	13	3	1	2	16	10	5	0	1	4
Non-Immune (2)	8	6	2	5	1	4	9	7	2	2	8	6	2	2	15	10	5	1	2	4

The actual average histogram values for each cluster are shown as a percentage, in the order immune, non-immune cluster.

### Similar IDD clusters share similar Pfam domains

In order to assess whether similar IDD clusters are associated with similar ordered domains, we examined the co-occurrence of Pfam domains [13] in each pair of similar clusters as defined in the previous sub-section. A co-occurrence was considered significant if the Pfam domain occurred in both the immune and non-immune clusters in a given pair. We also restricted our analysis to Pfam domains that occurred more than once in either the immune or non-immune sets. The frequency of each of the Pfam domains in its respective cluster was much higher than that expected by chance (see Methods). A total of 51 Pfam domains satisfied the above criteria (Additional File 1), and the most significant results are listed in Table 3.

**Table 3:** Co-occurrence of Pfam domains in IDD clusters

Immune	Obs./Expect	Non-immune	Obs./Expect	Short Name	Full Name	Function (Uniref ID)
1 (5/30)	2.54	18 (3/11)	3.12	SH2	SH2 domain	Signal transduction (P05480)
3 (3/11)	5.06	41 (2/20)	2.75	C1_1	Phorbol esters/diacylglycerol binding domain (C1 domain)	RNA binding & signal transduction (P97433)
6 (2/3)	16.90	18 (5/21)	2.72	Guanylate_kin	Guanylate kinase	Possible Signal transduction (B9EHJ3 )
6 (2/25)	2.03	18 (3/11)	3.12	bZIP_1	bZIP transcription factor	Transcription (P01101)
38 (3/25)	6.35	31 (3/40)	4.83	SH3_1	SH3 domain	Adaptor (Q64010)
72 (2/15)	5.00	55 (2/30)	2.53	7tm_1	7 transmembrane receptor (rhodopsin family)	GPCR (O09047)

100 (2/33)	7.27	38 (6/48)	2.88	zf-C2H2	Zinc finger, C2H2 type	Possible transcription regulator (Q8C687)
100 (2/10)	24.00	38 (3/10)	6.90	KRAB	KRAB box	Possible transcription regulator (Q8BIV1)

115 (1/5)	7.35	26 (2/6)	2.94	ARID	ARID/BRIGHT DNA binding domain	Possible transcription regulator (Q3U108)

175 (2/12)	12.50	2 (2/13)	5.16	fn3	Fibronectin type III domain	Putative neuronal cell adhesion molecule (Q8BQC3)

A subset of all 51 co-occurring Pfam domains is listed. Columns 1-2 and 3-4 describe the immune and non-immune clusters, respectively. The first number is the cluster index, and the numbers in parentheses are the number of occurrences of the Pfam domain of interest and the total number of Pfam domains in the cluster, respectively. The 2^nd ^and 4^th ^columns are the ratio of the observed to expected number of Pfam domains in the cluster of interest, where the expected number is given by eqn. 5.

**Figure 5:**
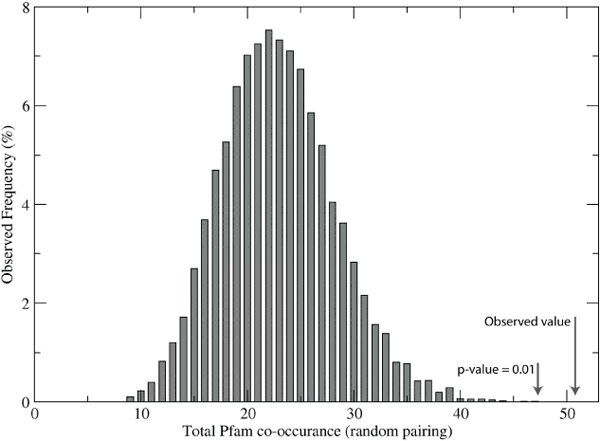
**Background distribution of Pfam domain co-occurrence**. Instead of using the Gaussian similarity score to match clusters in the innate immune set and the generic disordered set, we inserted a random matching function. The resulting distribution clearly indicates that the number of co-occurring Pfam domains identified by Gaussian similarity is highly significant.

In order to estimate the significance of the observed number of shared Pfam domains identified by the histogram similarity score, we replaced the maximization of the Gaussian similarity score (used to match clusters in the innate immune set with clusters in the Non-immune set) with a random pairing of clusters. There are many possible combinations of pairs, so we repeated the random pairing a total of 9000 times and obtained a background distribution of Pfam domain co-occurrence (figure 5). The maximum value in this exercise was 47, corresponding to a p-value of 0.01, based on direct integration of the frequency distribution. Therefore, we can say with a high degree of confidence that the co-occurrence of 51 Pfam domains is not due to chance (with a p-value << 0.01), and thus there is a bias for similar IDDs to be associated with specific Pfam domains.

The majority of the Pfam domains listed in Additional File 1 are involved in transcription, signal transduction, or both. The high IDD content in mammalian transcription factors has been examined before [14]. Thus it is difficult to make a simple functional interpretation of the IDD clusters. However, the co-occurrence of Pfam domains indicates that a frequency distribution-based analysis is practically useful for suggesting possible biological or biochemical roles of un-annotated IDDs. Although the number of co-occurring Pfam domains is higher than that expected by chance, there are, nevertheless, many Pfam domains that do not co-occur with specific IDD cluster with a high statistical significance.

## Discussion

**Figure 6:**
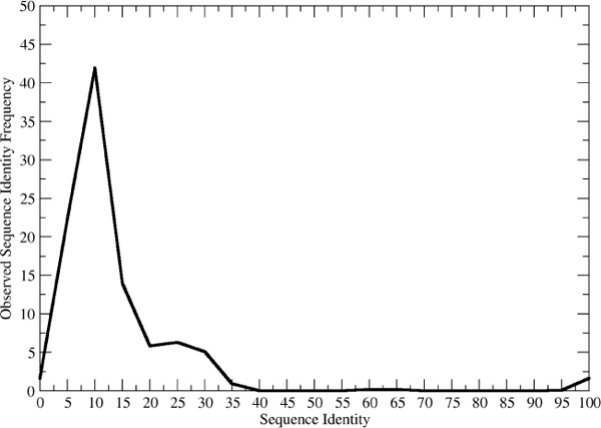
**Sequence identity within ordered folds**. The figure was constructed by picking 10 query domains at random, and calculating the sequence identity of all similar folds to the query as returned by the SeSAW structural alignment server [18].

In this study we carried out comparison of IDDs at both the overall amino acid composition level and at the local sequence motif level. These two levels of comparison span a wide range and yet we observe similar trends in both extremes. Namely, individual IDD sequences are very different from artificially constructed sequences picked naively. This, in turn, might imply that there is strong selective pressure on IDDs, just as there is strong pressure on ordered domains; however, direct evidence for this interpretation is beyond the scope of the current study. In the case of ordered domains we can understand such pressure in terms of the structural and functional requirements. The resulting distribution of ordered protein sequences is a trade-off between genetic drift, which tends toward randomization, and biochemical function, which tends to limit the observed amino acid sequences to a small subset of the possible random combinations. If we examine the distribution of sequence identities within a given fold, for example, we usually see two peaks (figure 6). One small peak is near 100% and contains the close family members. The other peak is broader and covers the "twilight zone" region from 0-30%. It is thus not unreasonable to hypothesize that a similar trade-off occurs for IDDs, and that the pressure in this case is due to the need for IDDs to be metastable, only becoming ordered upon binding a target protein. Understanding the exact role of specific IDDs will help to refine the interpretation of their compositional diversity.

In the case of local sequence motifs, we observed a strong bias toward low complexity patterns. These patterns did not include obvious binding site for kinases, SH2, or SH3. Therefore, it was not straightforward to assign a functional role to the motifs. However, the abundance of amino acid repeats in local sequence motifs in IDDs and the non-random nature of the IDDs together raise the possibility that the maintenance of these repeats may provide an additional restraint during the evolution of IDDs. Though the prevalence of amino acid repeats in disordered regions have been studied [15], the combined evolution of amino acid repeats and disordered regions needs to be investigated further. Moreover, our analysis did not reveal a systematic difference between the IDDs in the innate immune set and those in the generic IDD set.

Further, we also find that similar IDD clusters are associated with certain Pfam domains indicating possible functional roles for, and limitations on, the IDDs.

## Conclusions

There were two motivations for the current study. The first was that IDDs could be clustered into sub-groups in order to allow a more fine-tuned assignment than merely "IDD" when assigning domains to uncharacterized sequences. The second was to associate these clusters with certain ordered domains to facilitate future annotation of IDD-containing protein sequences. In terms of these goals, we were modestly successful as the histogram-based method is efficient and appropriate to classify IDDs according to their most abundant amino acids. With regard to the association with ordered domains, we were also successful, as judged by the statistical significance of Pfam domains associated with the innate immune and generic IDD data sets. In terms of practical importance, the IDD clusters identified here should be of use in characterizing orphan sequences containing IDDs. The classification of IDDs remains an open problem. One interesting avenue of future work will be to examine the predicted structural and functional constraints in IDD evolution.

## Methods

### Innate immune IDD set

Amino acid sequences corresponding to 1580 mouse genes potentially relevant to macrophage response to microbial stimulation were downloaded from the Innate Immune Database [16]. This list combines genes with significant expression changes under stimulation with lipopolysaccharides (LPS), and genes coding for proteins known to interact in the TNFα/NF-κB signaling pathway. A representative set of 1237 protein sequences was prepared using the cd-hit program [17] at 40% sequence identity. For each sequence disordered regions were predicted using the Disopred2 program [1] and 1464 predicted disordered regions of length 30 or more were retained for analysis.

### Non innate immune IDD set

Amino acid sequences 1663 human proteins were selected at random, excluding those in the Innate immune set, and a representative set of 2171 disordered regions of length 30 or more was prepared as above.

### Ordered set

The amino acid sequences for a set of 1999 representative structural domains was taken from Protein Data Bank (PDB) atom records.

### Random sequence sets

Randomized versions of each of the above 3 sequence sets were prepared as follows. The entire sequence set was concatenated into a single string and shuffled. The length of each of the original sequences was stored. Then for each of the original sequence lengths, a random sequence was constructed by repeatedly picking an amino acid at random from the concatenated sequence and transferring it to the random sequence. In this way, the resulting set of randomized sequences has the same length distribution and the overall amino acid composition was identical to that of the original sequence set.

### Similarity score for amino acid compositions

The frequency of a particular amino acid type *a *in an individual sequence *i *was given by the ratio of the number of *N(a) *to the length of the sequence

(1) $$f_{i}(a)=\frac{N(a)}{len_{i}}$$

The Gaussian similarity score for a pair of frequencies was given by

(2) $$Sim_{i,j}=\frac{\sum_{a=1}^{20}\left[f_{i}(a)+f_{j}(a)\right]e^{-{\left(\frac{f_{i}(a)-f_{j}(a)}{w}\right)}^{2}}}{\sum_{a=1}^{20}f_{i}(a)+f_{j}(a)}$$

The Gaussian term is always non-negative and evaluates to 1 for a perfect match between sequences *i *and *j*. The width of the Gaussian *w *is an adjustable parameter empirically set to 0.1 for the purpose of distributing most of the similarity values over the range 0.5-1. The exponential terms are weighted by the average frequency in order to give more emphasis to abundant amino acids. The denominator evaluates to 2 in every case so it is not actually necessary but we include it for completeness. Without this weighting term we found that zero counts were dominating the similarity score. Note that pseudo counts are not needed for the above score (i.e., for zero values in the histograms of short IDDs). For clustering, it is convenient to convert the similarity to a pseudo distance by

(3) $$Dist_{i,j}=1-Sim_{i,j}$$

We also generalized eqn. 2 for comparing clusters of sequences. We defined the distance between two amino acid frequencies in two clusters as the distance between their average values (|⟨*f*_*i*_(*a*)⟩ - ⟨*f*_*i*_(*a*)⟩|) for clusters *i *and *j*, and we set the square of the width of the Gaussian *w^2 ^*to the sum of the square of the standard deviations (*w*^2 ^= σ_*i*_(*a*)^2 ^+ σ_*i*_(*a*)^2^). With these modifications, the Gaussian score becomes

(4) $$ClustSim_{i,j}=\frac{\sum_{a=1}^{20}\left[\left\langle f_{i}(a)\right\rangle +\left\langle f_{j}(a)\right\rangle \right]e^{-\left({\frac{\left(\left\langle f_{i}(a)\right\rangle -\left\langle f_{j}(a)\right\rangle \right)}{\sigma_{i}{\left(a\right)}^{2}+\sigma_{j}{\left(a\right)}^{2}}}^{2}\right)}}{\sum_{a=1}^{20}\left\langle f_{i}(a)\right\rangle +\left\langle f_{j}(a)\right\rangle }$$

where again the denominator is included only for completeness.

### Expected Pfam domain co-occurrence

In order to access the significance of Pfam domain co-occurrence we computed the expected value for the number of Pfam domains *p *in cluster *c *as

(5) $$N_{\exp}^{p,c}=N_{tot}^{p}\frac{N^{c}}{\sum_{c}N^{c}}$$

where $N_{tot}^{p}$ is the total number of occurrences of Pfam domain *p *in the entire set of sequences, *N*^*c *^is the number of Pfam domains in cluster *c*, and the denominator is the sum of *N*^*c *^over all clusters (i.e., the total number of Pfam domains).

### Enumeration of short sequence motifs

Sequence motifs of length *n *were generated by explicitly enumerating all possible fragments. Given background amino acid frequencies *p(a) *the expectation value for a given motif was given by the product of the individual background frequencies and the total number of possible fragments in the dataset with given length *N(n)*.

(6) $$ev(a_{1}...a_{n})=N(n)\prod_{i}^{n}p(a_{i})$$

## Competing interests

The authors declare that they have no competing interests.

## Authors' contributions

ST carried out the local motif analysis, and evaluated the statistical methods. AP analyzed raw data and helped to draft the manuscript. DMS conceived of the study, carried out the amino acid compositional analysis and helped to draft the manuscript. All authors read and approved the final manuscript.

## Supplementary Material

Additional file 1 **Table S1 - Co-occurrence of Pfam domains in IDD clusters**. All 51 co-occurring Pfam domain are listed Columns 1-2 and 3-4 describe the immune and non-immune clusters, respectively. The first number is the cluster index, and the numbers in parentheses are the number of occurrences of the Pfam domain of interest and the total number of Pfam domains in the cluster, respectively. The 2^nd ^and 4^th ^columns are the ratio of the observed to expected number of Pfam domains in the cluster of interest, where the expected number is given by eqn 5. Click here for file
