# Supplementary material for: Intrinsically disordered domains deviate significantly from random sequences in mammalian proteins
Source: BMC Bioinformatics. 2010 Oct 15;11(Suppl 7):S7. doi: 10.1186/1471-2105-11-S7-S7 (PMC2957690; doi:10.1186/1471-2105-11-S7-S7)
Supplement: Additional file 1 — Table S1 - Co-occurrence of Pfam domains in IDD clusters. All 51 co-occurring Pfam domain are listed Columns 1-2 and 3-4 describe the immune and non-immune clusters, respectively. The first number is the cluster index, and the numbers in parentheses are the number of occurrences of the Pfam domain of interest and the total number of Pfam domains in the cluster, respectively. The 2nd and 4th columns are the ratio of the observed to expected number of Pfam domains in the cluster of interest, where the expected number is given by eqn 5. [file 1471-2105-11-S7-S7-S1.pdf]

## Additional file 1

**Intrinsically disordered domains deviate significantly from random sequences in mammalian proteins**

**by Shunsuke Teraguchi, Ashwini Patil and Daron M. Standley**

**Table S1 - Co-occurrence of Pfam domains in IDD clusters**

Columns 1-2 and 3-4 describe the immune and non-immune clusters, respectively. The first number is the cluster index, and the numbers in parentheses are the number of occurrences of the Pfam domain of interest and the total number of Pfam domains in the cluster, respectively. The 2nd and 4th columns are the ratio of the observed to expected number of Pfam domains in the cluster of interest, where the expected number is given by eqn. 5.

| Immune | Obs./ Expect | Non-immune | Obs./ Expect | Short Name | Full Name | Function (Uniref ID) |
|---|---|---|---|---|---|---|
| 0 (1/7) | 8.87 | 99 (3/20) | 6.80 | SAM_1 | SAM domain (Sterile alpha motif) | Lipid catabolic process (Q80Y98) |
| 0 (1/6) | 10.34 | 99 (2/8) | 11.34 | DDHD | DDHD domain | Lipid degradation (Q80Y98) |
| 1 (5/30) | 2.54 | 18 (3/11) | 3.12 | SH2 | SH2 domain | Signal transduction (P05480) |
| 1 (1/3) | 5.08 | 18 (2/4) | 5.71 | Exo_endo_phos | Endonuclease/Exonuclease/phosphatase family | Signal transduction (Q9ES52) |
| 1 (2/7) | 4.36 | 18 (1/3) | 3.81 | PAS_3 | PAS fold | Transcription & signal transduction (P97481) |
| 3 (3/11) | 5.06 | 41 (2/20) | 2.75 | C1_1 | Phorbol esters/diacylglycerol binding domain (C1 domain) | RNA binding & signal transduction (P97433) |
| 3 (2/4) | 9.28 | 41 (1/4) | 6.88 | PB1 | PB1 domain | Signal transduction (Q61084) |
| 6 (2/3) | 16.90 | 18 (5/21) | 2.72 | Guanylate_kin | Guanylate kinase | Possible Signal transduction (B9EHJ3 ) |
| 6 (2/25) | 2.03 | 18 (3/11) | 3.12 | bZIP_1 | bZIP transcription factor | Transcription (P01101) |

| Immune | Obs./ Expect | Non-immune | Obs./ Expect | Short Name | Full Name | Function (Uniref ID) |
|---|---|---|---|---|---|---|
| 6 (1/5) | 5.07 | 18 (3/15) | 2.28 | Hormone_recep | Ligand-binding domain of nuclear hormone receptor | Transcription (Q60644) |
| 6 (1/5) | 5.07 | 18 (3/15) | 2.28 | zf-C4 | Zinc finger, C4 type (two domains) | Transcription (Q60644) |
| 9 (1/3) | 5.00 | 23 (3/28) | 5.31 | CH | Calponin homology (CH) domain | Possible apoptosis/cell cycle (P11862) |
| 9 (2/7) | 4.29 | 23 (1/16) | 3.10 | Chromo | 'chromo' (CHRromatin Organisation MOdifier) domain | Transcription (Q09XV5) |
| 9 (2/6) | 5.00 | 23 (1/15) | 3.30 | BRK | BRK domain | Transcription (Q09XV5) |
| 12 (1/21) | 2.96 | 2 (5/27) | 6.21 | Pkinase_Tyr | Protein tyrosine kinase | Signal transduction (Q3UDE9) |
| 12 (1/13) | 4.77 | 2 (2/21) | 3.20 | WD40 | WD domain, G-beta repeat | Cholesterol metabolism (Q6GQT6 ) |
| 15 (2/23) | 4.01 | 126 (1/24) | 3.73 | HLH | Helix-loop-helix DNA-binding domain | Various (Q6GTZ3) |
| 29 (1/5) | 2.06 | 26 (2/7) | 2.52 | EGF | EGF-like domain | Transcription (O35516) |
| 29 (1/4) | 2.57 | 26 (2/4) | 4.41 | zf-DBF | DBF zinc finger | Cell cycle DNA replication (Q9QZ41) |
| 29 (1/5) | 2.06 | 26 (2/6) | 2.94 | Rhodanese | Rhodanese-like domain | Ubiquitin pathway (Q80U87) |
| 29 (1/2) | 5.14 | 26 (2/8) | 2.21 | SAP | SAP domain | DNA replair (P23475) |
| 29 (1/5) | 2.06 | 26 (2/6) | 2.94 | ARID | ARID/BRIGHT DNA binding domain | Transcription (Q62431) |
| 36 (1/3) | 17.14 | 30 (2/6) | 71.58 | Lectin_C | Lectin C-type domain | Unknown (Q3U3M1) |
| 36 (2/25) | 4.11 | 30 (1/40) | 5.37 | SH3_1 | SH3 domain | Cell cycle kinase binding (O35177) |
| 38 (1/25) | 2.12 | 31 (3/77) | 2.51 | PH | PH domain | Tyr kinase (Q03526) |
| 38 (3/25) | 6.35 | 31 (3/40) | 4.83 | SH3_1 | SH3 domain | Adaptor (Q64010) |
| 38 (4/30) | 7.06 | 31 (1/11) | 5.86 | SH2 | SH2 domain | Ubiquitination/signalling (Q8VHQ2) |
| 41 (1/12) | 2.31 | 38 (4/44) | 2.09 | Helicase_C | Helicase conserved C-terminal domain | Mda5 RNA helicase (Q8R5F7) |
| 41 (1/7) | 3.96 | 38 (2/20) | 2.30 | Bromodomain | Bromodomain | Possible transcription activator (Q7JJ13) |

| Immune | Obs./ Expect | Non-immune | Obs./ Expect | Short Name | Full Name | Function (Uniref ID) |
|---|---|---|---|---|---|---|
| 53 (1/1) | 43.90 | 26 (3/7) | 3.78 | SCAN | SCAN domain | Transcription (Q8CF60) |
| 56 (1/5) | 14.40 | 18 (3/15) | 2.28 | Hormone_recep | Ligand-binding domain of nuclear hormone receptor | Transcription (Q545Q1) |
| 56 (1/5) | 14.40 | 18 (3/15) | 2.28 | zf-C4 | Zinc finger, C4 type (two domains) | Transcription (Q545Q1) |
| 56 (1/3) | 24.00 | 18 (2/4) | 5.71 | Exo_endo_phos | Endonuclease/Exonuclease/phosphatase family | Circadian rhythm (O35710) |
| 56 (1/6) | 12.00 | 18 (2/10) | 2.28 | dDENN | dDENN domain | Possible Rab6-mediated GTPase signaling (Q6PAL8) |
| 56 (1/6) | 12.00 | 18 (2/10) | 2.28 | uDENN | uDENN domain | Possible Rab6-mediated GTPase signaling (Q6PAL8) |
| 56 (1/6) | 12.00 | 18 (2/10) | 2.28 | DENN | DENN (AEX-3) domain | Possible Rab6-mediated GTPase signaling (Q6PAL8) |
| 72 (1/6) | 6.25 | 55 (2/10) | 7.58 | dDENN | dDENN domain | Possible Rab6-mediated GTPase signaling (Q6PAL8) |
| 72 (1/6) | 6.25 | 55 (2/10) | 7.58 | uDENN | uDENN domain | Possible Rab6-mediated GTPase signaling (Q6PAL8) |
| 72 (1/6) | 6.25 | 55 (2/10) | 7.58 | DENN | DENN (AEX-3) domain | Possible Rab6-mediated GTPase signaling (Q6PAL8) |
| 72 (2/15) | 5.00 | 55 (2/30) | 2.53 | 7tm_1 | 7 transmembrane receptor (rhodopsin family) | GPCR (O09047) |
| 79 (1/17) | 2.41 | 26 (2/7) | 2.52 | Ets | Ets-domain | Transcription (Q8VDK3) |
| 79 (1/7) | 5.84 | 26 (2/4) | 4.41 | SAM_PNT | Sterile alpha motif (SAM)/Pointed domain | Transcription (Q8VDK3) |
| 80 (1/13) | 17.31 | 2 (2/21) | 3.20 | WD40 | WD domain, G-beta repeat | ER-Golgi transport (O55029) |
| 94 (1/12) | 3.26 | 42 (3/44) | 3.09 | Helicase_C | Helicase conserved C-terminal domain | RNA helicase DDx58 (Q6Q899) |
| 94 (4/16) | 9.78 | 42 (1/5) | 9.07 | RGS | Regulator of G protein signaling domain | Signal transduction (O08849) |

| Immune | Obs./ Expect | Non-immune | Obs./ Expect | Short Name | Full Name | Function (Uniref ID) |
|---|---|---|---|---|---|---|
| 100 (2/33) | 7.27 | 38 (6/48) | 2.88 | zf-C2H2 | Zinc finger, C2H2 type | Possible transcription regulator (Q8C687) |
| 100 (2/10) | 24.00 | 38 (3/10) | 6.90 | KRAB | KRAB box | Possible transcription regulator (Q8BIV1) |
| 107 (1/13) | 8.65 | 38 (2/21) | 2.19 | WD40 | WD domain, G-beta repeat | Transcription factor (Q8BL74) |
| 115 (2/17) | 4.32 | 26 (2/7) | 2.52 | Ets | Ets-domain | Transcription factor (Q60775) |
| 115 (1/5) | 7.35 | 26 (2/6) | 2.94 | ARID | ARID/BRIGHT DNA binding domain | Possible transcription regulator (Q3U108) |
| 175 (2/12) | 12.50 | 2 (2/13) | 5.16 | fn3 | Fibronectin type III domain | Putative neuronal cell adhesion molecule (Q8BQC3) |
